# Malaria and pre-eclampsia in an area with unstable malaria transmission in Central Sudan

**DOI:** 10.1186/1475-2875-10-258

**Published:** 2011-09-07

**Authors:** Ishag Adam, Elhassan M Elhassan, Ahmed A Mohmmed, Magdi M Salih, Mustafa I Elbashir

**Affiliations:** 1 Faculty of Medicine, University of Khartoum, P.O. Box 102, Khartoum, Sudan; 2 Faculty of Medicine, University of Gezira, P.O. Box 42, Medani, Sudan; 3 Faculty of Medicine, The National Ribat University, P.O. Box 408, Khartoum, Sudan; 4 Faculty of Medical Laboratory Sciences, University of Khartoum, P.O. Box 102, Khartoum Sudan

## Abstract

**Background:**

Placental malaria and pre-eclampsia occur frequently in women in tropics and are leading causes of maternal and perinatal morbidities and mortality. Few data exist concerning the interaction between placental malaria and pre-eclampsia.

**Methods:**

A case control study was conducted in Medani Hospital, which locates in an area of unstable malaria transmission in Central Sudan. Case (N = 143) were women with pre-eclampsia, which was defined as systolic blood presure≥140 mm Hg or diastolic blood pressure ≥ 90 mm Hg and proteinuria. Controls were parturient women (N = 143) without any blood pressure values > 139/89 mm Hg or proteinuria. Obstetrical and medical characteristics were gathered from both groups through structured questionnaires. Placental histopathology examinations for malaria were performed.

**Results:**

Twenty-eight (19.6%) vs. 16 (11.2%); *P *= 0.04 of the cases vs. controls, had placental malaria infections. Five (2%), 1 (2%) and 22 (28.0%) vs. 1, 2 and 13 of the placentae showed acute, chronic and past infection on histopathology examination in the two groups respectively, while 115 (80.4%) vs.127 (88.8%) of them showed no infection, *P *= 0.04. In multivariate analysis, while there were no associations between age, parity, educational level, lack of antenatal care, blood groups and body mass index and pre-eclampsia; family history of hypertension and placental malaria (OR = 2.3, 95% CI = 1.0-5.2; *P *= 0.04) were significantly associated with pre-eclampsia.

**Conclusion:**

Placental malaria was associated with pre-eclampsia. Further research is needed.

## Background

Pre-eclampsia, one of the most common medical complications of pregnancy, it affects approximately 10% of all human births [1]. It is a leading cause of maternal mortality worldwide, as well as an important cause of perinatal mortality [2]. It has been estimated that 90% of the global malaria burden occurs in sub-Saharan Africa, where during pregnancy 40% women are exposed to malaria infections [3]. Malaria during pregnancy poses a substantial risk to the mother, her fetus and the neonate [4]. Although pre-eclampsia and maternal malaria would be expected frequently to occur concurrently in malarious areas, their interaction on the health of the mother and her baby has been little studied [5]. Seasonal changes in the incidence of pre-eclampsia have been described in tropics, which are consistent with malaria transmission periods [6]. Placental malaria is likely to impair placental development and cause maternal hypertension and placental vascular dysfunction [7].

Few data exist concerning placental malaria and its association with pre-eclampsia. While some of these studies have reported the association between malaria and pre-eclampsia and malaria and hypertension during pregnancy [8,9], other studies failed to find a significant association between malaria and pre-eclampsia [10,11]. Furthermore some of these studies did not differentiate between pre-eclampsia and other types of hypertension.

Malaria during pregnancy is a major health problem in Sudan, where pregnant Sudanese women are more susceptible to malaria, and it is associated with maternal anaemia and poor maternal and perinatal outcomes [12-14]. Both malaria and pre-eclampsia were among the common causes of high maternal mortality in Sudan [15,16]. The current study was conducted in Wad Medani maternity hospital in central Sudan to investigate the association between placental malaria and pre-eclampsia, so as to add on the standing and the recent research on both placental malaria and pre-eclampsia in Sudan [17,18]. The area is characterized by unstable malaria transmission and *Plasmodium falciparum *is the sole malaria species in the area [19].

## Methods

### Patients

A case control study was conducted during the period of June 2009 through January 2010, at the labour ward of Medani Maternity Hospital, Central Sudan. Medani Maternity Hospital has a tertiary care for women who receive antenatal care at the hospital as well as for referrals from the other clinics and hospitals, and women who are close to the hospital facility. All women with risk factors or obstetric complications are referred to the hospital. The referral criteria are not strictly adhered to and many patients without any significant complications deliver at the hospital.

A case was defined as a woman who had given birth and who, in the antenatal period or before going into labour, was diagnosed as being pre-eclamptic. Pre-eclampsia was defined as pregnancy -induced hypertension associated with proteinuria. Pregnancy-induced hypertension was defined as new hypertension with blood pressure of 140 mm Hg systolic or diastolic blood pressure of 90 mm Hg diastolic or greater arising after 20 weeks of gestation in a woman who was normotensive before 20 weeks gestation. Proteinuria was defined as excretion of 300 mg or more of protein in 24 hour urine sample or ≥ 2+ or more on dipstick. A consecutive control was taken for each case. Controls were parturient women admitted for delivery, without any blood pressure values greater than 139/89 or proteinuria recorded in the pregnant health card during the antenatal visits and at the time of delivery. Two measures of blood pressure were done using a sphygmomanometer. Blood pressure measures were taken at the time of admission after a 10-minute rest and far from a uterine contraction; the second measure of blood pressure was taken between 30 and 60 minutes later in the same conditions. Proteinuria (albumin) was detected in a fresh urine sample before active labor by dipsticks (Albustix). Twins and diabetic women were excluded because these were known predictors for pre-eclampsia [20]. After signing an informed consent, from women in both case and control groups, socio-demographic, obstetrics and medical characteristics were gathered through structured questionnaires. Women were enquired for using bed nets and malaria infections in the index pregnancy. Body mass index was calculated by measuring maternal weight and height and expressed as weight (kg)/height (m) ^2^).

### Haematology

Maternal, placental and cord blood films were prepared, the slides were Giemsa-stained and the number of asexual *P. falciparum *parasites per 200 white blood cells was counted and double-checked blindly by an expert microscopist. Maternal blood groups were investigated by the agglutination method.

### Placental histology

Full thickness placental blocks of around 2-3 cm were taken from the placenta, kept in neutral buffer formalin for histopathology examinations. Placental malaria infections were characterized based on the classification of Bulmer *et al *[21]: uninfected (no parasites or pigment), acute (parasites in intervillous spaces), chronic (parasites in maternal erythrocytes and pigment in fibrin or cells within fibrin and/or chorionic villous syncytiotrophoblast or stroma), past (no parasites and pigment confined to fibrin or cells within fibrin).

### Statistics

Data were entered in computer using SPSS for windows version 16.0. Means and proportions were compared by Students' *t*-test, × ^2 ^and Fisher's exact tests as appropriate. Univariate and multivariate analyses were performed where pre-eclampsia as a dependent variable and maternal socio-demographic characteristics (age, parity and maternal blood group, past history and family history of pre-eclampsia) and placental malaria as possible influencing factors. *P *≤ 0.05 was regarded as significant.

### Ethics

The study received ethical clearance from the Research Board at the Faculty of Medicine, University of Khartoum.

## Results

During the study period, there were 4,620 deliveries. Of these, 160 had pre-eclampsia. One hundred and forty-three pre-eclamptic women had complete data including placental histology and were included in the final analyses. These data were compared with equal number of controls with complete data, Table 1. In comparison to the controls, significantly higher numbers of cases were primigravidae, had history of malaria infections in the index pregnancy and had family history of hypertension, Table 1. There was no significant difference in the mean (SD) age [28.9 (6.2) vs. 28.4(6.0) years], and body mass index [24.4 (2.2) vs.25.0 (3.5) kg/m^2^] in preeclamptic vs. controls women. The coverage of bed nets was low in the two groups, Table 1.

**Table 1:** Socio-demographic and malaria state in the cases and controls

*Number (%) of *	Pre-eclamptic (*N *= 143)	Controls (*N *= 143)	*P*
Primigravidae	49(34.3)	27(18.9)	0.01
lack of antenatal care	21(14.7)	33(23.1)	0.02
Educational level < secondary	36(25.2)	24(16.8)	0.8
Bednets coverage	21(14.7)	23 (16.1)	0.6
History of malaria	31(21.7)	11(7.7)	0.001
Family history of hypertension	122(85.3)	81(56.6)	0.001
Placental malaria	28(19.6)	16(11.2)	0.04

Maternal and placental blood films for malaria were positive in one of the cases and one of the controls had positive placental blood film. Twenty-eight (19.6%) vs. 16 (11.2%); *P *= 0.04 of the cases vs. controls, had placental malaria infections. Five (2%), 1 (2%) and 22 (28.0%) vs. 1, 2 and 13 of the placentae showed acute, chronic and past infection on histopathology examination in the two groups respectively, while 115 (80.4%) vs.127 (88.8%) of them showed no infection, *P *= 0.04.

### Predictors for pre-eclampsia in univariate and multivariate analysis

There were no associations between the age (OR = 1.0, 95 CI = 0.9-1.1; *P *= 0.1), educational level (OR = 1.0, 95% CI = 0.5-2.0; *P *= 0.1) blood groups (OR = 1.1, 95% CI = 0.6-2.0; *P *= 0.5) and body mass index (OR = 1.0, 95% CI = 0.9-1.1; *P *= 0.9) and pre-eclampsia, Table 2.

**Table 2:** Factors associated with pre-eclampsia in Medani Maternity hospital, Central Sudan using univariate or multivariate analysis

	Univariate analysis			Multivariate analysis		
**The variable**	**OR**	**95% CI**	***P***	**OR**	**95% CI**	***P***

Age	1.0	0.9-1.0	0.5	1.0	0.9-1.1	0.1
Primigravidae	2.5	1.5-4.4	0.001	0.9	0.7-1.0	0.2
Family history of hypertension	6.7	3.7-12.2	0.001	5.7	2.9-11.5	0.001
History of malaria	3.7	1.8-7.7	0.001	2.0	0.9-4.4	0.07
Educational level < secondary	0.9	0.9-1.0	0.3	1.0	0.5-2.0	0.9
Lack of antenatal care	2.0	1.0-3.6	0.02	2.4	0.9-6.1	0.06
Body mass index	0.9	0.8-1.0	0.3	1.0	0.9-1.1	0.9
Blood group O vs.non group O	1.4	0.9-2.3	0.08	1.1	0.6-2.0	0.5
Placental malaria	1.9	1.0-3.7	0.05	2.3	1.0-5.2	0.04

Primigravidae, lack of antenatal care and history of malaria in the index pregnancy were associated with pre-eclampsia in univariate analysis only. In multivariate analysis family history of hypertension (OR = 5.7, 95% CI = 2.9-11.5; *P *= 0.001) and placental malaria (OR = 2.3, 95% CI = 1.0-5.2; *P *= 0.04) were significantly associated with pre-eclampsia, Table 2.

## Discussion

The main findings of the current study were; there was no significant association between age, educational level, body mass index and blood group and pre-eclampsia. While women with family history of hypertension and those who had placental malaria infections were at higher risk of pre-eclampsia in multivariate analyses, primigravidae, lack of antenatal care and history of malaria in the index pregnancy were associated with pre-eclampsia in univariate analysis only. Recently, age, parity, body mass index were the risk factors for pre-eclampsia observed among Latin American and Caribbean women [20].

In the current study women who had placental malaria infections were twice at higher risk for pre-eclampsia (OR = 2.3, 95% CI = 1.0-5.2; *P *= 0.04) compared with women without pre-eclampsia. This is consistent with the previous observation in Senegal with a 3.3-fold increase in the risk of placental malaria infections within women with diastolic blood pressure greater than or equal to 90 mm Hg compared with controls, where it was less than 90 mm Hg [8]. The difference between the current study and the later one is the classification of hypertensive women according to the presence or absence of significant proteinuria in the current study. Pre-eclampsia has also been shown to be more common in Senegal during the rainy season [22]. Likewise, in Tanzania, young first-time mothers with placental malaria had significantly increased risk of hypertension [23]. Interestingly, recently Ndao *et al *[9] observed association between placental malaria infections (using placental histology as diagnostic tool) and non-proteinuric hypertension in women living in a malaria-hypoendemic area in Senegal. Yet they did not find an association between placental malaria and pre-eclampsia [9]. However in neighbouring Kenya studies failed to find a relationship between malaria during pregnancy and pre-eclampsia [10,11].

The majority of the placental infections in the current study were past infections.

There is a critical phase in early/mid pregnancy when transformation of the spiral arteries should take place, which is critical in pathogenesis of future pre-eclampsia.

Massive sequestration of parasites in the placenta leads to placental ischemia and loss of placental integrity, increases production of pro-inflammatory cytokines, and increases endothelial dysfunction [24,25]. Moreover, it has recently been observed that the production/imbalance of cytokines as pathophysiologic mechanisms of pre-eclampsia and placental malaria infections in two different studies [17,18]. In 2006 in Tanzania, Muehlenbachs *et al *[23] have suggested that maternal-foetal conflict involving the inflammatory mediator, vascular endothelial growth factor pathway occurs during placental malaria and that its inhibitor, soluble vascular endothelial growth factor receptor 1 may be involved in a possible relation between chronic placental malaria infections and hypertension in primigravidae. However, it might still not be clear which the cause of effect is.

## Conclusion

Family history of hypertension and placental malaria were associated with pre-eclampsia. Larger longitudinal studies are needed.

## Competing interests

The authors declare that they have no competing interests.

## Authors' contributions

IA and MIE coordinated, carried out the study and participated in the statistical analysis and procedures, EME participated in the clinical work and statistical analysis. AM and MS participated in the lab work. All the authors read and approved the final version.
